# Cytokine responses during chronic denervation

**DOI:** 10.1186/1742-2094-2-26

**Published:** 2005-11-18

**Authors:** Saku Ruohonen, Mohsen Khademi, Maja Jagodic, Hanna-Stiina Taskinen, Tomas Olsson, Matias Röyttä

**Affiliations:** 1 Department of Pathology, University of Turku, Kiinanmyllynkatu 10, 20520 Turku, Finland; 2 Department of Neuroscience, Karolinska Institute, 17176 Stockholm, Sweden; 3 Department of Handsurgery, Turku University hospital, Kiinanmyllynkatu 10, 20520, Turku, Finland

## Abstract

**Background:**

The aim of the present study was to examine inflammatory responses during Wallerian degeneration in rat peripheral nerve when the regrowth of axons was prevented by suturing.

**Methods:**

Transected rat sciatic nerve was sutured and ligated to prevent reinnervation. The samples were collected from the left sciatic nerve distally and proximally from the point of transection. The endoneurium was separated from the surrounding epi- and perineurium to examine the expression of cytokines in both of these compartments. Macrophage invasion into endoneurium was investigated and Schwann cell proliferation was followed as well as the expression of cytokines IL-1β, IL-10, IFN-γ and TNF-α mRNA. The samples were collected from 1 day up to 5 weeks after the primary operation.

**Results:**

At days 1 to 3 after injury in the epi-/perineurium of the proximal and distal stump, a marked expression of the pro-inflammatory cytokines TNF-α and IL-1β and of the anti-inflammatory cytokine IL-10 was observed. Concurrently, numerous macrophages started to gather into the epineurium of both proximal and distal stumps. At day 7 the number of macrophages decreased in the perineurium and increased markedly in the endoneurium of both stumps. At this time point marked expression of TNF-α and IFN-γ mRNA was observed in the endo- and epi-/perineurium of the proximal stump. At day 14 a marked increase in the expression of IL-1β could be noted in the proximal stump epi-/perineurium and in the distal stump endoneurium. At that time point many macrophages were observed in the longitudinally sectioned epineurium of the proximal 2 area as well as in the cross-section slides from the distal stump. At day 35 TNF-α, IL-1β and IL-10 mRNA appeared abundantly in the proximal epi-/perineurium together with macrophages.

**Conclusion:**

The present studies show that even during chronic denervation there is a cyclic expression pattern for the studied cytokines. Contrary to the previous findings on reinnervating nerves the studied cytokines show increased expression up to 35 days. The high expressions of pro-inflammatory and anti-inflammatory cytokines in the proximal epi-/perineurial area at day 35 may be involved in the formation of fibrosis due to irreversible nerve injury and thus may have relevance to the formation of traumatic neuroma.

## Background

During Wallerian degeneration, macrophages enter the peripheral nervous system [1-3] when permeability of the BNB is increased. The increased permeability of BNB leads to the increased infiltration of macrophages into the endoneurium [4,5]. Injury-activated Schwann cells and resident macrophages are probably partly responsible for the recruitment of hematogenous macrophages by secreting MCP-1 [6] and the pro-inflammatory cytokines IL-1, IL-6, IL-12; and especially TNF-α, in the early phase of the inflammatory reaction [7-13]. The main population of macrophages in the peripheral nerve is blood-derived after injury. These gather in the endoneurium to start phagocytosis of axons and myelin stumps [14,15]. Macrophages and Schwann cells mediate and promote inflammation after injury by production of pro-inflammatory cytokines, but they have also positive effects on neurotrophic factors, which can induce axonal sprouting [16]. Macrophages as well as Schwann cells express also anti-inflammatory cytokines such as IL-10 and TGF-β1 to inhibit inflammation [17-21]. The role of TGF-β1 during nerve injury seems to be controversial. It increases neuronal regeneration [19,22] but decreases NGF production [23-25] and kills Schwann cells together with TNF-α [26].

In the present study permanent nerve damage was induced by suturing the distal and proximal ends of the transected sciatic nerve in order to create a model of chronic denervation. Since relatively small amounts of cytokine mRNAs are present in the nerve stumps, real-time polymerase chain reaction was used to facilitate detection of the studied cytokines. Additionally, morphological differences were followed to investigate possible correlations with produced cytokines.

## Materials and methods

### Experimental animals

Young adult male Sprague-Dawley rats (n = 70) were used in the present study. The animals were kept in the Turku University Animal Centre. (Circadian 12-h rhythm, T = 21 ± 1°C, humidity 50 ± 5%)

Normal daily care was provided with nutrition (Chow Lactamin R36, Södertälje, Sweden) and water ad libitum. The present study was approved by the Committee for Ethical Animal Experiments (permission no. 1080/01).

### Operative procedures

The left sciatic nerves were exposed and transected at the level of hip joint under pentobarbital (Mebunat^®^) anesthesia. Regeneration at the left sciatic nerve was prevented with suturing of distal and proximal stumps beside the point of transection [27]. The right sciatic nerve was left intact. Additionally, normal control nerve samples were collected from normal, unoperated rats of the same age. Samples were collected 1 day, 3 days, 5 days, 7 days; and 2, 3, 4 and 5 weeks after the primary operation. For the subsequent biochemical studies six rats were sacrificed at each time point, and two at each time point for the immunohistochemical studies. The sciatic nerves of three normal rats were studied as negative controls. The rats were perfused intracardially with sterile 0.9% saline or with 4% phosphate-buffered formalin. The nerves were cut in 4-mm sections in freezing conditions (on a covered Petri dish filled with ice).

### Real-time PCR and histological samples

**Figure 1:**
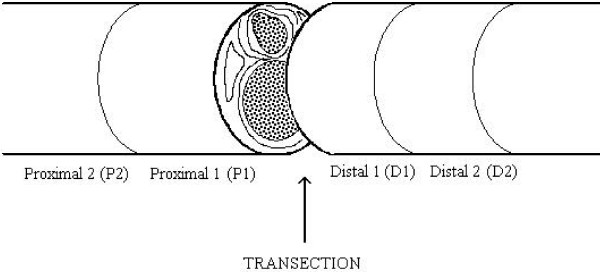
**Transection point**. Left sciatic nerves were exposed and transected at the level of the hip joint under pentobarbital anaesthesia. Regeneration of the left sciatic nerve was prevented by suturing of distal and proximal stumps beside the point of transection. The right sciatic nerve was left intact. For biochemical and morphological analysis two 4-mm areas (P1, P2) were cut starting 1 mm proximally to the point of transection. Also, distally two sections were cut, one (D1) starting immediately 1 mm from the point of transection and the other (D2) starting 5 mm distal to the site of transection.

**Figure 2:**
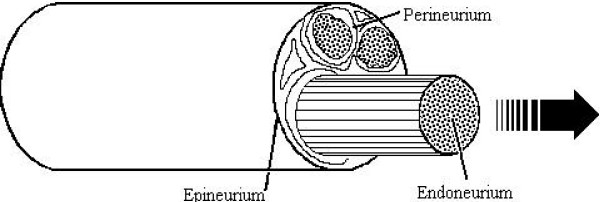
**Separation of endo-and epi-/perineurium**. For biochemical studies, endoneurium was separated from the surrounding peri- and epineurium. This was done on the icy cover of a Petri dish filled with ice. The endoneurium was pulled out with fine tip forceps.

The samples were taken both proximally and distally from the point of transection. To avoid local damage and/or sutures beside the point of transection, the initial 1-mm section beside the point of transection was discarded. For real-time PCR and immunohistochemical analysis two 4-mm sections (P1, P2) were cut starting 1 mm proximally from the point of transection. Also, distally two sections were cut, one (D1) starting immediately 1 mm from the point of transection and the other (D2) starting 5 mm distally from the point of transection (Fig. 1). Furthermore, for the real-time PCR studies, the endoneurium was separated from the surrounding peri- and epineurium (Fig. 2) [28]. All real-time PCR samples were immediately immersed in a GITS solution, frozen to -196°C with liquid nitrogen, and stored at -70°C. The samples were pooled from six different rats for each time point to obtain more homogenous material for the PCR studies.

### Immunohistochemistry

The animals for morphological samples were perfusion-fixed with 4% phosphate-buffered formalin and whole peripheral nerve samples were exposed and fixed overnight in 4% phosphate-buffered formalin. The specimens were embedded in paraffin and 4-μm sections were cut for immunohistochemical analyses. The paraffin sections were treated with xylene and decreasing alcohol solutions to remove paraffin, and then hydrated and digested with 0.4% pepsin in 0.01 M HCl for 60 min. Endogenous peroxidase activity was blocked using 0.3% H_2_O_2 _in methanol, after which sections were incubated for 60 min with horse serum to prevent non-specific staining. The sections were then treated with monoclonal antibody ED-1 (Serotec 0591) for monocytes/macrophages or S-100 (Dakopatts) for Schwann cells overnight at 4°C. Bound antibodies were demonstrated using an avidin-biotin method with a Vectastain ABC kit according to the manufacturer's instructions. Additionally, normal rat serum was used with the second antibody to diminish non-specific staining.

The recruitment of macrophages was evaluated microscopically. Semi-quantification of changes was performed as in our previous studies [1,29]. In the endoneurium the visual evaluation of macrophages was done with a scale of four: 0 = only occasional macrophages similar to that seen in the control nerve; + = a few macrophages, no focal accumulation of macrophages; ++ = several macrophages with focal accumulation, phagocytotic activity present; +++ = numerous macrophages with phagocytotic activity. Similar principles were used in the epineurium. However, this evaluation was done with a scale of three: 0 = only occasional macrophages similar to that seen in the control nerve; + = a few macrophages per cross section in the epineurium; ++ = over 10 macrophages per cross-section in the epineurium.

Schwann cells were assessed using similar techniques. However, the proliferation pattern of Schwann cells was similar to that found in previous studies [29]. Therefore, the data from Schwann cells is not shown.

### Immunofluorochemisty

For immunofluorochemical studies veins of animals were stained with FITC-Dextran (Sigma, FD2000S) iv-injection. The amount of FITC-Dextran was titrated to 100 mg/kg to show the veins of the sciatic nerve in a whole-mount experiment. Thirty minutes after injection the animals were perfused with saline and 4% phosphate-buffered formalin. Samples were taken distally and proximally from the point of transection. The nerve stumps were fixed overnight in 4% phosphate-buffered formalin and then transferred to 70% EtOH. The nerve stumps were cut into four longitudinal sections before washing with TTBS and Triton-X permeabilization. BSA (1%) was used to prevent non-specific staining. The sections were then treated with monoclonal antibody ED-1 (1:200; Serotec 0591) 2 h at 37°C. Bound antibodies were demonstrated using goat-anti-mouse-Alexa 555 antibody (1:200; Molecular Probes). The numbers of ED-1 positive cells were compared to that observed in control nerve from unoperated rats, using cross-sectional samples from the same area. The samples were studied by confocal microscope (LSM 510, Zeiss Axiovert 200).

### Determination of cytokine mRNA from endo- and epi/perineurium

Pooled and frozen nerve stumps were first homogenized with Ultra-Turrax. The RNA preparation was modified from Chomczynski and Sacchi [30]. RNA was extracted using Trizol (Gibco BRL 15596-018) according to the acid phenol-quanidium thiocyanate-chloroform extraction method. mRNA was purified from total RNA, and then reverse-transcribed to cDNA. The reverse transcription was performed using 10 μg of mRNA, and Superscript reverse transcriptase (200 U; Life Technologies). The samples were then measured with real-time PCR.

cDNA amplification was performed with the ABI PRISM 7700 Sequence Detector (Applied Biosystems) with a two-step PCR protocol (preincubation 10 min at 95°C followed by 40 cycles at 95°C for 15 sec and at 60°C for 1 min). All cytokine primers and probes were designed using Primer Express software (Applied Biosystems) avoiding contaminating genomic DNA amplification by positioning one of the primers or a probe over the exon/intron boundary. The probes were labeled with FAM at the 5' end as reporter dye and TAMRA at the 3' end as quencher dye. The GAPDH gene was used as a stable endogenous control.

Absolute quantification was performed using a standard curve method. Standard curves were constructed using three 100-fold dilutions of standard sample (for IL-1β the standards were plasmids containing the corresponding cytokine gene; for GAPDH, TNF-α and IL-10 the standards were cDNA from concanavalin-A stimulated rat lymph node cells) and corresponding cycles of threshold (Ct) value. The samples were run in triplicate for target cytokine and endogenous GAPDH controls. After computing the relative amounts of target cytokine and endogenous control for one sample, the final amount of cytokine in that sample was presented as a ratio between and the amount of cytokine and amount of endogenous control, GAPDH. For real-time PCR, quantitative results are available directly after PCR without additional purification or analysis steps. Protein levels of cytokines were not studied.

The results were imported into Microsoft Excel and legends were made with GraphPad Prism. SEM was calculated with one-way ANOVA and t-test, which were made with Microcal Origin. The source SEM does not reflect differentiation between individual animals but methodological accuracy.

## Results

### Results of real-time PCR studies

#### IL-1β (Figure 3.)

**Figure 3:**
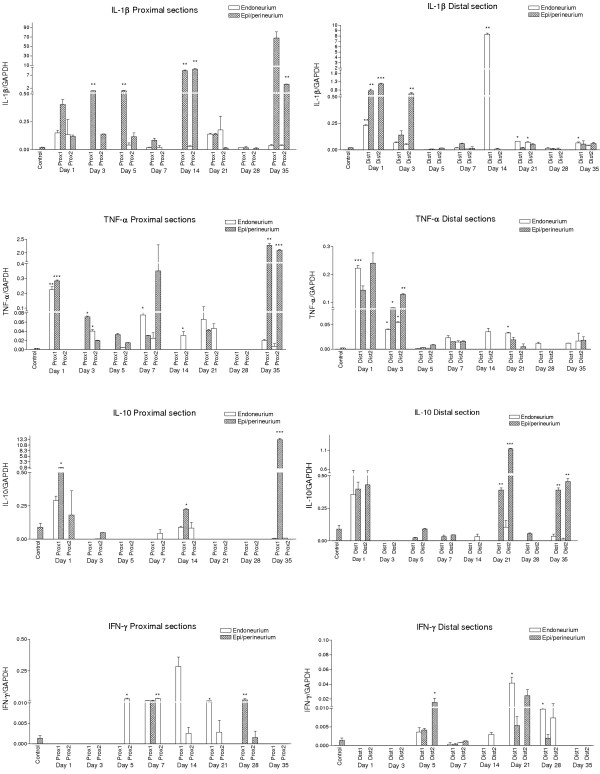
**Cytokine expression**. Cytokine expressions of IL-1β, IL-10, IFN-γ and TNF-α after transection. Statistical analyses were made with one-way ANOVA and t-test. Only those expressions that had t-values > 0 (relative to control expression) were taken into account. Significance values are marked as follows: * p = 0.05; ** p = 0.01; *** p = 0.001. The expression changes of different cytokines mRNAs could be observed at multiple time points after peripheral nerve injury. Expressions in control samples could only be observed in the epi-/perineurium. The proximal stump expressed IL-1β, TNF-α and IL-10 in a cyclic manner. The main time points for the cyclic expression pattern were 1-5, 14-21 and 35 days. The distal stump showed marked expression of these cytokines during days 1-3 after injury, but for cytokine IL-1β also at day 14. IL-10 was markedly expressed at 21 and 35 days after injury. IFN-γ was not expressed in either compartment before day 5. The proximal stump showed marked expression of this cytokine at 14 days after injury and the distal stump at 21 days. IL-4 was not markedly expressed in this study.

##### Controls

Very low expression of IL-1β mRNA was observed only in the epi/perineurium of the control samples.

##### Proximal areas

In the endoneurium the strongest expressions of IL-1β mRNA were observed in both proximal areas (P1 and P2) at 24 hours and at day 21 after injury. At other time points the expression remained low.

The epi/perineurium expressed IL-1β mRNA in a cyclic manner in proximal areas. Three expression peaks were observed: the first one during days 3 and 5, the second one at day 14 (proximal 2) and the third and strongest one at day 35. Proximal 2 areas showed lower expression of cytokines than the proximal 1 area.

##### Distal areas

The endoneurium showed a slight increase in the expression of IL-1β starting from day 1 until day 7 after injury. The expression rose again dramatically (110-fold) at day 14. Otherwise the expression of IL-1β mRNA was low.

In the epi/perineurium marked expressions were observed during days 1 and 3 in both distal areas. At the other time points the expression stayed at the control level.

#### TNF-α (Figure 3.)

##### Controls

Very low TNF-α mRNA expression was observed in the epi/perineurium but not in the endoneurium.

##### Proximal areas

The endoneurium showed strong TNF-α mRNA expression in the first proximal area at 24 hours. After that only small peaks of expression were noted at days 7, 14, 21 and 35.

The epi/perineurium expressed TNF-α mRNA in a fashion similar to that of the endoneurium. The first proximal areas epi/perineurium showed marked expression of TNF-α at 24 hours. However, the most pronounced expression was noted at day 35 in the proximal 2 area.

##### Distal areas

In the endoneurium the strongest expression of TNF-α mRNA was noted during the first days after injury but was at the control level at day five. The expression rose slightly at days 7, 14 and 21 in both distal areas, after which only low expression was observed.

In the epi/perineurium the expression pattern of TNF-α mRNA followed the pattern observed in the endoneurium but with increased expressions. From day 5, the expression stayed at the control level.

#### IFN-γ (Figure 3.)

##### Controls

Very low expression of IFN-γ mRNA was observed only in the epi/perineurial control samples but not in the endoneurium.

##### Proximal areas

The endoneurium did not show IFN-γ mRNA until day 5 after which it declined. The most marked expression of IFN-γ mRNA was noted at day 14 after injury.

In the epi-/perineurium only the first proximal area showed some peaked expression of IFN-γ mRNA and only at days 7 and 28 after injury.

##### Distal areas

The endoneurium showed marked expression of IFN-γ mRNA in the distal 1 area at day 21, but some expression was also noted at day 28 in both distal stumps. Also some expression was noted at days 5 and 14.

The epi/perineurium showed increased expression of IFN-γ only at days 5 and 21 after injury.

#### IL-10 (Figure 3.)

##### Controls

Only slight IL-10 mRNA expression was noted in the epi-/perineurium of the operated sciatic nerves but not in the endoneurium.

##### Proximal areas

The endoneurium showed increased expression of IL-10 at 24 hours and at days 7 and 14 after injury.

In the epi/perineurium IL-10 mRNA was expressed already at 24 hours but the most marked expression was seen at day 35 (900-fold) compared to controls after injury.

##### Distal areas

The endoneurium showed a marked increase in expression only at 24 hours. Slight expression was noted at days 14, 21 and 35.

The epi/perineurium showed marked peaks of expression at days 1, 21 and 35; otherwise the expression remained at the control level.

### Results of morphological studies

#### Macrophages (Figures 4A,B,C,D,E,F)

**Figure 4:**
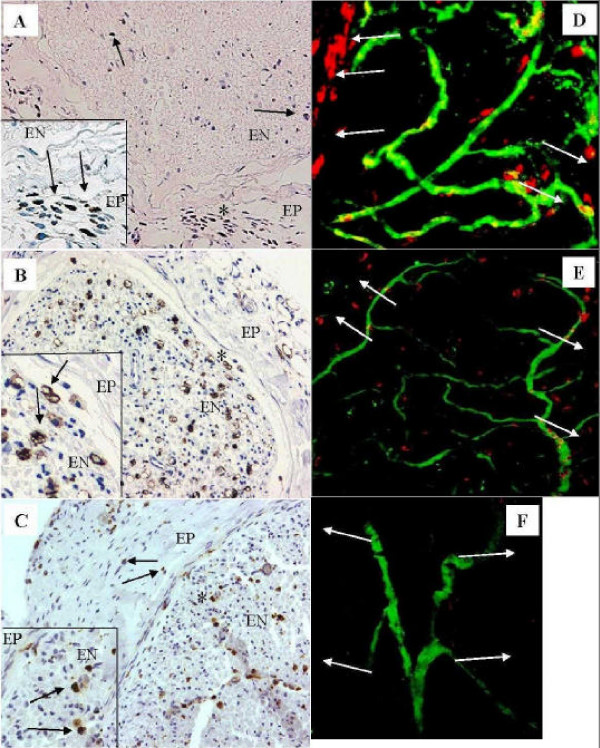
**Macrophages**. **(A-C) **The areas marked with asterisk (*) are shown with a higher magnification (×420). Black arrows indicate macrophages. (A) At day 3 macrophages are present in the epineurium (EP) of distal 1 area (×210). A few ED-1 positive cells are also seen in the endoneurium (EN). (B) In distal 1 area at day 14 there are numerous macrophages in the endoneurium (×210). (C) Some macrophages are still present in both epi- and endoneurium in the proximal 2 area at 35 days (× 210). **(D-F) **Longitudinal sections studied by confocal microscope. Macrophages are visualized with red color, endoneurial vessels with green, and yellow color indicates macrophages inside blood vessels. White arrows indicate the epineurial area. The pictures are focused to the endoneurial level in the middle. (D) At day 14 several macrophages can be observed in the epineurial and endoneurial area of proximal 2 area (× 120). (E) At day 35 several macrophages are still present in the epineurium of proximal 2 area, but start to decrease in the endoneurium (× 120). (F) Control from sciatic nerve (non-operated control animal) (× 120).

In the distal and proximal stump the epineurial macrophages could be observed at day 3, (++) (Fig. 4A). Invasion of macrophages into the endoneurial space took place at days 5 and 7 in the proximal and distal stumps, (++) [1]. At days 14 (Fig. 4B) and 21 several macrophages were observed in the distal endo- and epineurium, (endoneurium +++; epineurium ++). At this time point the proximal stump seemed to have only a few MHC II-expressing cells in cross-section samples (+). However, confocal-microscopically studied longitudinal sections of proximal 2 area revealed focal accumulation of macrophages in the epineurial area (Fig. 4D) compared to normal control nerves (Fig. 4F). After this time point only a few macrophages were observed in the distal stump. However, occasional macrophages could still be observed in the proximal and distal endo- (++) and epineurium (+) at day 35 in cross-sections (Fig. 4C), as well as in the longitudinal samples studied by confocal microscope (Fig. 4E).

## Discussion

To achieve a model for a chronic nerve injury, and in an attempt to provoke marked and long-lasting immunological changes, transected sciatic nerve was sutured and ligated to prevent reinnervation. In order to better understand the possible dynamics in different compartments within the peripheral nerve, the endoneurium was separated from the surrounding epi/perineurium (Fig. 2) [28] and these compartments were analyzed separately. We have previously shown that after peripheral nerve transection a marked number of macrophages appear first in the epineurium, after which they enter the endoneurium [1]. Hence, the expression of cytokines may differ in these peripheral nerve compartments due to expression of cytokines such as IL-1β and TNF-α, which affect vascular permeability [31] and lead to increased infiltration of macrophages into the endoneurial space during Wallerian degeneration. Our previous studies on peripheral nerve degeneration and regeneration [29,32,33] indicate that when nerve stumps are sutured to prevent regeneration, the most prominent source for endoneurial later-phase expression of cytokines could be Schwann cells and possibly fibroblasts. These cell types are significantly present during the later time points when monocytes/macrophages are already almost absent in the endoneurium. The main source for marked pro-inflammatory cytokine expression in the epi-/perineurium would be macrophages. But, mast cells may also present a potent source for cytokines in the peripheral nervous system [34].

The main sources of IL-1β after peripheral nerve injury are macrophages and Schwann cells [35,36]. In the present study macrophages were numerous in the proximal perineurium showing simultaneous high expression of IL-1β mRNA in the epi-/perineurium at days 1-5. The expression of IL-1β rose to a peak at days 14 and 35 after injury in the epi-/perineurium. At day 14 in the epineurial cross-section samples macrophages were almost absent. However, in the longitudinal samples studied by confocal microscope, focal accumulation of macrophages was seen in this particular area. The distal stump showed marked expression of IL-1β in the endoneurium at day 14 and at this time point numerous macrophages as well as Schwann cells were present in the distal endoneurium.

We have previously observed that, after classical Wallerian degeneration and regeneration, TNF-α mRNA is upregulated in the distal areas from 14 hours to day 5 and at day 14. In the proximal areas TNF-α is expressed from 14 hours to day 1 and again at day 5 [32]. The present results with sutured nerves showed a similar pattern of expression. TNF-α mRNA expression increased rapidly at 24 hours both in the endo- and epi/perineurium of distal and proximal areas. TNF-α has several inflammation-mediating capabilities during peripheral nerve injury [37-40]. Thus, marked rapid activation is needed for stimulating inflammatory reactions involving macrophages [41,42]. This includes the infiltration of macrophages into the endoneurium when BNB is broken down [43] at the beginning of Wallerian degeneration [44] where TNF-α shows pathogenic actions [8]. We found a mild increase in TNF-α mRNA expression at day 14 in the distal area. This augmentation of expression could be related to increased vascular permeability at two weeks [5,31]. Also, the number of Schwann cells starts to decrease at this time point [29] and thus an explanation for this distal TNF-α mRNA expression at 14 days could be the ability of TNF-α to induce Schwann cell apoptosis [45]. In the present study, however, the expression of TNF-α in the distal stump was relatively much lower at day 14 compared to our previous studies on freely regenerative nerves. This indicates that the arrival of axons to the distal stump would also be a possible starter of apoptosis of Schwann cells and hence, an inducer of expression of TNF-α in the distal area. The most remarkable TNF-α mRNA expressions were noted at day 35 in the epi-/perineurium of the proximal area 2. This peak of expression has not been observed in freely regenerative nerve models [32,46]. The reason for this is not known.

In the present study the expression of IL-10 mRNA increased rapidly in proximal and distal sections at 24 hours after injury both in the endo- and epi/perineurium, most likely as an inhibitory response to inflammatory reactions that TNF-α and IL-1β had provoked [17,47,48]. The source for IL-10 expression at the very beginning after injury cannot be infiltrating macrophages [49] because these cells are not usually observed before day 3 in the endoneurium. The most probable source at this time would be Schwann cells [20]. However, the resident activated macrophages that are normally present in the endoneurium are also activated during the inflammation [7,9] and may be responsible for the observed expression. Infiltrating hematogenous macrophages are probably partially responsible for the high IL-10 expression noted at day 21 in the distal epi-/perineurium, and in the endoneurium this expression would be augmented by Schwann cells [20,36]. An interesting observation was that from day 1 onwards the expression of IL-10 mRNA was mainly found in both distal and proximal epi/perineurium. At day 28 the expression of IL-10 was minimal in the distal epi-/perineurium. However, despite the low expression of TNF-α, IL-1β and IL-10 at this time point, the distal stump presents several morphological findings [27,50]. Endoneurial fibroblasts, which are markedly present in this area, form minifascicle-like structures, which possibly support basal lamina tubes from collapsing [27]. Interestingly the expression of IL-10 mRNA was markedly increased at day 35 in distal and proximal epi/perineurium. These changes could be related to the noted simultaneous high expressions of the two pro-inflammatory cytokines (IL-1β and TNF-α). IL-10 is known to inhibit the production of these inflammation-related cytokines [17,49,51]. IL-10 diminishes apoptosis provoked by TNF-α [45] and down-regulates MHC class II expression in macrophages/monocytes [49]. The producers of IL-10 during this later phase are at least partially macrophages [20,49], which were simultaneously present with IL-10 expression in the epineurium in the present study. However, mast cells [52] are also capable of producing IL-10. Why this expression of cytokines in the epi/perineurium takes place at day 35 is unknown. The present experimental design, with prevention of axonal reinnervation, may provoke these changes. Thus one cannot exclude the possibility that the observed expression at day 35 may be related to structural reorganization of peripheral nerve compartments.

In the present study IFN-γ mRNA was present after day 5. This observation can be temporally linked to macrophages, which enter the endoneurium [1,32]. In neuroinflammatory processes IFN-γ, which is primarily released by T-lymphocytes, has been noted to have an important role as an upregulator of MHC class II antigen expression [53]. IFN-γ increases influx of T cells and macrophages into the peripheral nervous system [54], and increases TNF-α and IL-1 production in macrophages [55] and fibroblasts [56]. By this action IFN-γ would induce a massive infiltration of macrophages into endoneurium 3 to 5 days after injury. The marked expression of IFN-γ mRNA at day 14 in the proximal endoneurium is also the time point at which a marked invasion of endoneurial blood-derived macrophages occurs in the proximal stump (Figure 4B.)

At day 35 several cytokines showed augmented expressions in the proximal 2 area in the present study. When axonal regeneration is prevented by suturing the peripheral nerve, a traumatic neuroma starts to form. A component of this neuroma is epineurial fibrosis, which is increased by TGF-β1 [57]. The expression of TGF-β1 increases dramatically in the proximal 2 area epineurium at day 35 during neuroma formation [58]. In the present study, a similar increase in expression of cytokines IL-1β, IL-10 and TNF-α was observed. This finding has not been reported previously.

When axonal sprouts start to grow in the proximal stump, some of them could accidentally start to grow backwards in the epineurium. However, these axons do not extend this growth very far, and sprouting is rapidly disabled. The disabled growth of axons in the epineurium has not been clarified but TGF-β1 is of special interest in this case. It has a function as a mitogen for Schwann cells [59] but it is also capable of killing them by activating c-Jun [60]. Activation of c-Jun leads also to increased expression of TNF-α, which is also capable of inducing Schwann cell apoptosis [26] as well as neuronal death [45,61].

Interestingly, non-injured contralateral sciatic nerve shows marked endoneurial expression of cytokines IL-1β, IL-10 and TNF-α mRNAs at day 35 after injury [62]. However, at this same time marked expressions of these cytokines at the site of injury were now observed only in the epi-/perineurium. What is the mechanism behind these paradoxical findings? This observation could indicate that the observed changes in expression of cytokines at the site of injury can spread through humoral stimulus via circulation. Activated macrophages have also been observed in the endoneurium of the contralateral side after injury [9]. Could the observed endoneurial changes in the contralateral nerve be a consequence of macrophage stimulation from the ganglions, brain, medulla or hypothalamus? After chronic constriction injury, blocking of NMDA-receptor was found to eliminate mRNA cytokine expression in contralateral sciatic nerve [63]. The biological significance of this finding is unknown.

## Conclusion

Our results show that prolonged inflammatory mediator changes take place in peripheral nerve in a chronic denervation injury model. The expression is cyclic and correlates partly with those noted in the non-injured contralateral sciatic nerve. This study also supported previous suggestions that Schwann cells may have a central role in the expression of cytokines in nerve [36], which could be partly responsible for pain behavior as well as for support of neural growth during neuroma formation. This denervation model offers new and interesting ways to study the pathogenesis of traumatic neuroma and neuroinflammatory changes, some of which may be related to pain.

## List of abbreviations

IL Interleukin

IFN-γ Interferon-γ

BNB Blood-nerve-barrier

BSA Bovine serum albumin

GAPDH Glyseraldehyde-3-phosphate-dehydrogenase

GITS Guanidine thiocyanate 6-9277 Sigma, N-lauroylsarcosine L-5125 Sigma, tri-Na-citrate dihydrate 1.12005 Merck, 0.7% h-mercaptoethanol

MHC Major histocompatibility complex

NGF Nerve growth factor

TGF-β1 Transforming growth factor-β1

NMDA N-methyl-D-aspartate

## Competing interests

The author(s) declare that they have no competing interests.

## Authors' contributions

SR carried out the real-time PCR studies, the histological studies, statistics, performed the animal operations together with MR and HST, and prepared the manuscript. MK, MJ and TO performed the real-time PCR analysis, as well as the preparation of mRNA samples. HST participated in the design and coordination of the study. MR designed the study, carried the responsibility for the study protocol and aided in the preparation of the manuscript.

**Table 1:** List of primers and probes used in the present study

**Cytokine**	**5' primer**	**3' primer**	**Probe**
**GAPDH**	TCAACTACATGGTCTACATGTTCCAG	TCCCATTCTCAGCCTTGACTG	TGACTCTACCCACGGCAAGTTCAACG
**IFN-γ**	TCGAATCGCACCTGATCACTA	GGGTTGTTCACCTCGAACTTG	CATCCTTTTTTGCTTTACTGTTGCTGAGAAG
**IL-1β**	GAAAGACGGCACACCCACC	AAACCGCTTTTCCATCTTCTTCT	TGCAGCTGGAGAGTGTGGATCCCAAAC
**IL-10**	CCCTCTGGATACAGCTGCG	GCTCCACTGCCTTGCTTTTATT	CGCTGTCATCGATTTCTCCCCTGTGA
**TNF-α**	GACCCTCACACTCAGATCATCTTCT	ACGCTGGCTCAGCCACTC	TAGCCCACGTCGTAGCAAACCACCAA

The probes were labeled with FAM in the 5' end and TAMRA in the 3' end.

## References

[B1] Taskinen HS, Röyttä M (1997). The dynamics of macrophage recruitment after nerve transection. Acta Neuropathol (Berl).

[B2] Stoll G, Griffin JW, Li CY, Trapp BD (1989). Wallerian degeneration in the peripheral nervous system: participation of both Schwann cells and macrophages in myelin degradation. J Neurocytol.

[B3] Perry VH, Brown MC, Gordon S (1987). The macrophage response to central and peripheral nerve injury. A possible role for macrophages in regeneration. J Exp Med.

[B4] Mellick RS, Cavanagh JB (1968). Changes in blood vessel permeability during degeneration and regeneration in peripheral nerves. Brain.

[B5] Olsson Y (1966). Studies on vascular permeability in peripheral nerves. I. Distribution of circulating fluorescent serum albumin in normal, crushed and sectioned rat sciatic nerve. Acta Neuropathol (Berl).

[B6] Toews AD, Barrett C, Morell P (1998). Monocyte chemoattractant protein 1 is responsible for macrophage recruitment following injury to sciatic nerve. J Neurosci Res.

[B7] Oldfors A (1980). Macrophages in peripheral nerves. An ultrastructural and enzyme histochemical study on rats. Acta Neuropathol (Berl).

[B8] Stoll G, Jung S, Jander S, van der Meide P, Hartung HP (1993). Tumor necrosis factor-alpha in immune-mediated demyelination and Wallerian degeneration of the rat peripheral nervous system. J Neuroimmunol.

[B9] Mueller M, Leonhard C, Wacker K, Ringelstein EB, Okabe M, Hickey WF, Kiefer R, eds (2003). Macrophage response to peripheral nerve injury: the quantitative contribution of resident and hematogenous macrophages. Lab Invest.

[B10] Shamash S, Reichert F, Rotshenker S (2002). The cytokine network of Wallerian degeneration: tumor necrosis factor-alpha, interleukin-1alpha, and interleukin-1beta. J Neurosci.

[B11] Wagner R, Myers RR (1996). Schwann cells produce tumor necrosis factor alpha: expression in injured and non-injured nerves. Neuroscience.

[B12] Sommer C, Schafers M (1998). Painful mononeuropathy in C57BL/Wld mice with delayed wallerian degeneration: differential effects of cytokine production and nerve regeneration on thermal and mechanical hypersensitivity. Brain Res.

[B13] George A, Buehl A, Sommer C (2004). Wallerian degeneration after crush injury of rat sciatic nerve increases endo- and epineurial tumor necrosis factor-alpha protein. Neurosci Lett.

[B14] Beuche W, Friede RL (1984). The role of non-resident cells in Wallerian degeneration. J Neurocytol.

[B15] Griffin JW, George R, Lobato C, Tyor WR, Yan LC, Glass JD (1992). Macrophage responses and myelin clearance during Wallerian degeneration: relevance to immune-mediated demyelination. J Neuroimmunol.

[B16] Asakura K, Rodriguez M (1998). A unique population of circulating autoantibodies promotes central nervous system remyelination. Mult Scler.

[B17] Fiorentino DF, Zlotnik A, Mosmann TR, Howard M, O'Garra A (1991). IL-10 inhibits cytokine production by activated macrophages. J Immunol.

[B18] Kiefer R, Streit WJ, Toyka KV, Kreutzberg GW, Hartung HP (1995). Transforming growth factor-beta 1: a lesion-associated cytokine of the nervous system. Int J Dev Neurosci.

[B19] Scherer SS, Kamholz J, Jakowlew SB (1993). Axons modulate the expression of transforming growth factor-betas in Schwann cells. Glia.

[B20] Jander S, Pohl J, Gillen C, Stoll G (1996). Differential expression of interleukin-10 mRNA in Wallerian degeneration and immune-mediated inflammation of the rat peripheral nervous system. J Neurosci Res.

[B21] O'Keefe GM, Nguyen VT, Benveniste EN (1999). Class II transactivator and class II MHC gene expression in microglia: modulation by the cytokines TGF-beta, IL-4, IL-13 and IL-10. Eur J Immunol.

[B22] Sulaiman OA, Gordon T (2002). Transforming growth factor-beta and forskolin attenuate the adverse effects of long-term Schwann cell denervation on peripheral nerve regeneration in vivo. Glia.

[B23] Rogister B, Delree P, Leprince P, Martin D, Sadzot C, Malgrange B, Munaut C, Rigo JM, Lefebvre PP, Octave JN (1993). Transforming growth factor beta as a neuronoglial signal during peripheral nervous system response to injury. J Neurosci Res.

[B24] Einheber S, Hannocks MJ, Metz CN, Rifkin DB, Salzer JL (1995). Transforming growth factor-beta 1 regulates axon/Schwann cell interactions. J Cell Biol.

[B25] Hattori A, Iwasaki S, Murase K, Tsujimoto M, Sato M, Hayashi K, Kohno M (1994). Tumor necrosis factor is markedly synergistic with interleukin 1 and interferon-gamma in stimulating the production of nerve growth factor in fibroblasts. FEBS Lett.

[B26] Skoff AM, Lisak RP, Bealmear B, Benjamins JA (1998). TNF-alpha and TGF-beta act synergistically to kill Schwann cells. J Neurosci Res.

[B27] Röyttä M, Salonen V, Peltonen J (1987). Reversible endoneurial changes after nerve injury. Acta Neuropathol (Berl).

[B28] Siironen J, Sandberg M, Vuorinen V, Roytta M (1992). Laminin B1 and collagen type IV gene expression in transected peripheral nerve: reinnervation compared to denervation. J Neurochem.

[B29] Siironen J, Collan Y, Roytta M (1994). Axonal reinnervation does not influence Schwann cell proliferation after rat sciatic nerve transection. Brain Res.

[B30] Chomczynski P, Sacchi N (1987). Single-step method of RNA isolation by acid guanidinium thiocyanate-phenol-chloroform extraction. Anal Biochem.

[B31] Schnell L, Fearn S, Klassen H, Schwab ME, Perry VH (1999). Acute inflammatory responses to mechanical lesions in the CNS: differences between brain and spinal cord. Eur J Neurosci.

[B32] Taskinen HS, Olsson T, Bucht A, Khademi M, Svelander L, Röyttä M (2000). Peripheral nerve injury induces endoneurial expression of IFN-gamma, IL-10 and TNF-alpha mRNA. J Neuroimmunol.

[B33] Taskinen HS, Röyttä M (2000). Increased expression of chemokines (MCP-1, MIP-1alpha, RANTES) after peripheral nerve transection. J Peripher Nerv Syst.

[B34] Burd PR, Thompson WC, Max EE, Mills FC (1995). Activated mast cells produce interleukin 13. J Exp Med.

[B35] Bergsteinsdottir K, Kingston A, Mirsky R, Jessen KR (1991). Rat Schwann cells produce interleukin-1. J Neuroimmunol.

[B36] Al-Shatti T, Barr AE, Safadi FF, Amin M, Barbe MF (2005). Increase in inflammatory cytokines in median nerves in a rat model of repetitive motion injury. J Neuroimmunol.

[B37] Liefner M, Siebert H, Sachse T, Michel U, Kollias G, Bruck W (2000). The role of TNF-alpha during Wallerian degeneration. J Neuroimmunol.

[B38] Sommer C, Schmidt C, George A, Toyka KV (1997). A metalloprotease-inhibitor reduces pain associated behavior in mice with experimental neuropathy. Neurosci Lett.

[B39] Wagner R, Myers RR (1996). Endoneurial injection of TNF-alpha produces neuropathic pain behaviors. Neuroreport.

[B40] Schafers M, Geis C, Brors D, Yaksh TL, Sommer C (2002). Anterograde transport of tumor necrosis factor-alpha in the intact and injured rat sciatic nerve. J Neurosci.

[B41] Nawroth PP, Stern DM (1986). Modulation of endothelial cell hemostatic properties by tumor necrosis factor. J Exp Med.

[B42] Misawa R, Kawagishi C, Watanabe N, Kobayashi Y (2001). Infiltration of neutrophils following injection of apoptotic cells into the peritoneal cavity. Apoptosis.

[B43] Tracey KJ, Cerami A (1994). Tumor necrosis factor: a pleiotropic cytokine and therapeutic target. Annu Rev Med.

[B44] Redford EJ, Hall SM, Smith KJ (1995). Vascular changes and demyelination induced by the intraneural injection of tumour necrosis factor. Brain.

[B45] Viviani B, Corsini E, Galli CL, Marinovich M (1998). Glia increase degeneration of hippocampal neurons through release of tumor necrosis factor-alpha. Toxicol Appl Pharmacol.

[B46] Okamoto K, Martin DP, Schmelzer JD, Mitsui Y, Low PA (2001). Pro- and anti-inflammatory cytokine gene expression in rat sciatic nerve chronic constriction injury model of neuropathic pain. Exp Neurol.

[B47] Bethea JR, Nagashima H, Acosta MC, Briceno C, Gomez F, Marcillo AE, Loor K, Green J, Dietrich WD (1999). Systemically administered interleukin-10 reduces tumor necrosis factor-alpha production and significantly improves functional recovery following traumatic spinal cord injury in rats. J Neurotrauma.

[B48] Perrin FE, Lacroix S, Aviles-Trigueros M, David S (2005). Involvement of monocyte chemoattractant protein-1, macrophage inflammatory protein-1{alpha} and interleukin-1{beta} in Wallerian degeneration. Brain.

[B49] Moore KW, O'Garra A, de Waal Malefyt R, Vieira P, Mosmann TR (1993). Interleukin-10. Annu Rev Immunol.

[B50] Salonen V, Aho H, Röyttä M, Peltonen J (1988). Quantitation of Schwann cells and endoneurial fibroblast-like cells after experimental nerve trauma. Acta Neuropathol (Berl).

[B51] Fiorentino DF, Zlotnik A, Vieira P, Mosmann TR, Howard M, Moore KW, O'Garra A (1991). IL-10 acts on the antigen-presenting cell to inhibit cytokine production by Th1 cells. J Immunol.

[B52] Bullock ED, Johnson EM (1996). Nerve growth factor induces the expression of certain cytokine genes and bcl-2 in mast cells. Potential role in survival promotion. J Biol Chem.

[B53] Halloran PF, Autenried P, Ramassar V, Urmson J, Cockfield S (1992). Local T cell responses induce widespread MHC expression. Evidence that IFN-gamma induces its own expression in remote sites. J Immunol.

[B54] Heininger K, Schafer B, Hartung HP, Fierz W, Linington C, Toyka KV (1988). The role of macrophages in experimental autoimmune neuritis induced by a P2-specific T-cell line. Ann Neurol.

[B55] Collart MA, Belin D, Vassalli JD, de Kossodo S, Vassalli P (1986). Gamma interferon enhances macrophage transcription of the tumor necrosis factor/cachectin, interleukin 1, and urokinase genes, which are controlled by short-lived repressors. J Exp Med.

[B56] Murwani R, Armati P (1998). Peripheral nerve fibroblasts as a source of IL-6, TNFalpha and IL-1 and their modulation by IFNgamma. J Neurol Sci.

[B57] Davison SP, McCaffrey TV, Porter MN, Manders E (1999). Improved nerve regeneration with neutralization of transforming growth factor-beta1. Laryngoscope.

[B58] Taskinen HS, Ruohonen S, Jagodic M, Khademi M, Olsson T, Roytta M (2004). Distinct expression of TGF-beta1 mRNA in the endo- and epineurium after nerve injury. J Neurotrauma.

[B59] Ridley AJ, Davis JB, Stroobant P, Land H (1989). Transforming growth factors-beta 1 and beta 2 are mitogens for rat Schwann cells. J Cell Biol.

[B60] Parkinson DB, Dong Z, Bunting H, Whitfield J, Meier C, Marie H, Mirsky R, Jessen KR (2001). Transforming growth factor beta (TGFbeta) mediates Schwann cell death in vitro and in vivo: examination of c-Jun activation, interactions with survival signals, and the relationship of TGFbeta-mediated death to Schwann cell differentiation. J Neurosci.

[B61] Raivich G, Liu ZQ, Kloss CU, Labow M, Bluethmann H, Bohatschek M (2002). Cytotoxic potential of proinflammatory cytokines: combined deletion of TNF receptors TNFR1 and TNFR2 prevents motoneuron cell death after facial axotomy in adult mouse. Exp Neurol.

[B62] Ruohonen S, Jagodi M, Khademi M, Taskinen HS, Ojala P, Olsson T, Roytta M (2002). Contralateral non-operated nerve to transected rat sciatic nerve shows increased expression of IL-1beta, TGF-beta1, TNF-alpha, and IL-10. J Neuroimmunol.

[B63] Kleinschnitz C, Brinkhoff J, Sommer C, Stoll G (2005). Contralateral cytokine gene induction after peripheral nerve lesions: dependence on the mode of injury and NMDA receptor signaling. Brain Res Mol Brain Res.

